# Editorial: Rising stars in comparative and clinical medicine: 2021

**DOI:** 10.3389/fvets.2022.1030960

**Published:** 2022-12-13

**Authors:** Fazul Nabi, Muhammad Asif Arain

**Affiliations:** ^1^Faculty of Veterinary and Animal Science, Lasbela University of Agriculture, Water and Marine Science, Uthal, Balochistan, Pakistan; ^2^Department of Traditional Chinese Veterinary Medicine, College of Veterinary Medicine, Southwest University, Chongqing, China

**Keywords:** gene expression, animal nutrition, poultry, microbiota, animal health, microbiology

The authors have been invited to serve as guest editors for this Research Topic. In this capacity, it was our pleasure to review a wide range of fascinating manuscripts and reviews within the field. In this editorial, we summarize the key findings presented in the Research Topic. The primary objective of this Research Topic is to provide a platform to share current research findings in the domain of comparative and clinical medicine. In the current era of emerging drug resistance and incalculable side effects of synthetic medicines (particularly antibiotics), this topic has been attracting increasing attention among researchers and the scientific community, who have been engaging in efforts to identify alternative biological compounds or drugs with the potential to promote health in multiple ways alongside limited side effects. Therefore, the researchers who have published articles under this Research Topic summarize a range of new evidence regarding the use of natural compounds with medicinal properties as an alternative to antibiotics in various species of livestock in order to improve the productive performance, immune functioning, and health status of animals. Numerous research questions and suggestions have thus been proposed as part of this Research Topic by rising stars in clinical medicine. The majority of studies published in this collection can be categorized into the following research areas: (1) animal health and management; (2) genetics and gene regulation; (3) microbiota in health and disease; and (4) poultry nutrition and morphology.

## Animal health and management

The livestock sector plays a vital role in providing animal food containing high-quality protein, essential nutrients, minerals, vitamins, and biological compounds to fulfill the physiological and nutritional requirements of the ever-rising population of human beings. Generally, a healthy animal can be defined as one that shows normal physiological behavior and is free from abiotic and biotic forms of infection that may alter the normal maintenance of homeostasis and normal physiology (1). Recently, the livestock and poultry sector has faced numerous challenges related to the input costs of production, global warming, heat stress, drug resistance, and the emergence of associated health problems (2, 3). A variety of nutritional strategies have been applied in animal feeding practices in domestic animals to optimize productive performance, improve health status, stimulate immune functions, and reduce the chances of infection (4). Infectious diseases and global warming are the major obstacles threatening the health and welfare of animals throughout the world. In the current era of modern science, several strategies have been developed to overcome these challenges, such as dietary supplementation of nutraceuticals, use of natural antioxidants in feeding practices, mineral supplementation, application of medicinal plants, derivatives, and genetic improvements (5, 6). The livestock sector is a subsector of agriculture that has not only played a central role in fulfilling the nutritional needs of the growing human population, but also contributed significantly to the economic growth of rural communities in general and national economies in particular (2). Recent development and growth in the livestock sector, particularly in developing countries, has been driven by several factors, including the growing human population, urbanization, its role as a source of employment and income, and the increasing availability of resource-efficient technology for modern farming systems. Over the last few decades, the potential productivity of livestock has been constantly improved by genetic modulation, nutritional management, environmentally controlled farming practices, disease prevention practices, and the use of antibiotic growth promoters. Moreover, future improvement of productive performance in this sector will require in-depth molecular research to explore the genetic potential of animals in terms of immunization and disease prevention. Additionally, further *in vitro* and *in vivo* research into alternative treatment options is needed in order to validate existing results on the use of these approaches for the treatment of infectious disease, with limited side effects, in order to resolve the emerging issue of antibiotic resistance. In this special issue, several of the researchers introduce novel research proposals in field of clinical and comparative medicine that might hold future benefits for researchers, scientists, and livestock farmers.

## Genetics and gene regulation

Recently, researchers have focused on gene therapy, in the process of which they have discovered novel pharmacotherapeutic targets in domains including gene cloning, identification, and expression, as well as the molecular signaling pathways controlling gene expression and functionality. The study of nutrigenomics has recently become an emerging area of research among the scientific community for exploration of the biological role of nutrients and other biological compounds in triggering or downregulating the functions of particular genes, leading to enhancements to immunological function in animals (7). Various nutrients have been incorporated into animal and poultry feed to promote the health status of the animals and of gene-related food products (meat, eggs, and milk). Niu et al. provide an overview of drug resistance mechanisms of the *Mycoplasma bovis* (M. bovis) pathogen in yaks that have arisen due to indiscriminate use of antibiotics, resulting in base mutations in drug target genes. Their results indicate that *M. bovis* in yaks exhibits single-site base mutations and two-base mutations leading to the production of strains highly resistant to aminoglycosides (genes rrs3 and rrs4) and fluoroquinolones (genes gyrA and parC). Furthermore, Wei et al. present an excellent review summarizing the underlying mechanism of cytotoxicity caused by fluoride poisoning. Their result suggest that fluorosis causes a series of changes associated with mitochondrial dysfunction, such as the generation of reactive oxygen species (ROS), cessation of the mitochondrial respiratory chain, mitochondrial fission, autophagy apoptosis, and mitochondrial calcium regulation. Gong et al. screened out the fat deposition genes in pigs and discovered that back fat deposition and thickness are associated with the expression of genes ACACA, SLC_2_A_4_, and THRSP in Tibetan pigs, while in the case of Yorkshire pigs, the genes associated with fat deposition are IDL_1_, ACACA, ELOVL_5_, PLAC_8_, SLC_2_A_4_, and THRSP. They conclude that signaling pathways and gene expression significantly affect fat deposition in both species of pig. Furthermore, several molecular techniques have been employed in clinical and comparative medicine for the diagnosis and treatment of diseases, and for drug and vaccine production, in order to improve animal productivity. Nabi et al. (8) suggest that the genotype and phenotype structures of all domestic animals can be impaired by continuous exposure to certain toxins, pathogens, and a variety of other compounds; these contribute to the liberation of certain enzymes and the production of free radicals of ROS, leading to oxidative stress and initiation of an inflammatory process alongside the development of pathological conditions. Meanwhile, the utilization of emerging technology for the development of alternative diagnostic and treatment options has been proposed, as a way to target the particular genes relevant to a given pathogen to improve the potential productivity of livestock animals.

## Microbiota in health and disease

The gastrointestinal tract (GIT) of animals is occupied by millions of microorganisms; these are collectively known as the gut microbiota and play a pivotal role in the animal body under normal circumstances. Several types of gut microbiota are found in the animal body, such as bacteria, fungi, protozoa, viruses, parasites, and archaea (Chen et al.). The animal body serves as a home for numerous microorganisms that may contribute to the function of communication between external and internal environments. Interestingly, one study published in this special issue reveals the involvement of the gut microbiota community in the development of mastitis in buffalo (Chen et al.). The authors of this article characterize the various bacterial and fungal communities in healthy and mastitis-affected animals and suggest that neither fungal nor bacterial activity is influenced by mastitis, with exceptions for a few bacteria and fungi. Similarly, Li et al. investigated the microbial diversity of the GIT in healthy and diarrheic horses. The findings of this study reveal that alpha diversity among GIT microorganisms declines significantly in diseased horses; however, several genera are dominant in the microbial community of both healthy and diseased animals.

Recent developments in the field of biotechnology and molecular biology have introduced numerous novel techniques such as gene sequencing and gene regulation, which support researchers in moving toward an in-depth understanding of the complexity and diversity of microbial populations in the animal body. Molecular phylogenetic analysis also provides details on the microbial community present in the GIT (9). It has been proven that the gut microbiota plays a significant role in several biological processes, such as stimulation of the metabolism, maintenance of energy balance, triggering of immunological responses, control of inflammatory processes, involvement in systemic diseases, initiation of neurological disorders, and making a contribution to obesity and host life processes (10). In conclusion, further research is needed to explore the connections between the host and the gut microbiota for treatment and disease prevention.

## Poultry nutrition and morphology

Poultry farming has expanded continuously over the last three decades, owing to its ability to supply protein-rich, high-quality meat and eggs at cheap prices to satisfy the growing demands of the human population. The poultry sector constitutes 37% of the world meat industry according to an OECD/FAO survey (2019), and there has been speculation that this sector will grow sharply in the coming years to produce about 331 million tons of meat in 2028 (4). This remarkable progress could be achieved through the application of advanced management practices, use of sub-therapeutic doses of AGPs (antibiotic growth promoters), control of infections, genetic improvement, and utilization of resource-efficient technologies (11). Currently, several nutritional strategies have been adopted to increase the production performance of egg- and meat-type poultry; these include nutritional manipulation with a number of anti-antibiotic feed additives, such as herbs and their extracts, and the use of nutraceuticals, probiotics, prebiotics, and immunostimulants, which are regarded as efficient and safe for use in poultry production systems (12).

In modern livestock and poultry farming practices, the extensive use of AGPs has been banned in several countries due to the emergence of antibiotic resistance and the transfer of this issue from the animal to the human domain. Therefore, research attention has been diverted to the identification of alternative compounds derived from natural sources that yield similar benefits with minimal side effects. In this context, medicinal plants and their biological compounds are of interest as a replacement for AGPs (13). In this Research Topic, Li et al. investigated the health-promoting potential in poultry birds of capsaicin alkaloid derived from capsicum fruit. The results of this study suggest that capsaicin alkaloid significantly improves the production performance of poultry birds by improving their metabolic efficiency, leading to greater organ weight and higher organoleptic quality of the broiler meat.

On this Research Topic, another study reveals the effect of capsaicin on the production performance of poultry. Similarly, another study also reports on the beneficial application of glycyrrhiza polysaccharide (GPS) in a poultry model, observing improved performance in terms of gut development and disease prevention (Wu et al.). The results show that dietary supplementation of GPS significantly enhances gut health by upregulating the expression of genes and cytokine production, leading to activation of T cells (CD4 and CD8) that may contribute to the maintenance of immune functions and the gut microbial community. Interestingly, Zhu et al. studied the role of telocyte cells (TCs) in the regulatory functions of the utero–vaginal junction (UVJ) and in intercellular communication in chickens. The authors of this study successfully demonstrate the presence of TCs at the UVJ of egg-laying chickens and speculate that these cells might play a role in maintaining the animals' physiological functions *via* intracellular communication and transfer of information related to sperm storage and oviduct infections. Overall, it can be concluded that the use of alternative nutritional strategies, in the form of dietary supplementation of phytobiotics, nutraceuticals, and immunostimulants, could be a preferable option to enhance the productive performance of various poultry species.

## Conclusion

Taken together, the articles published in this Research Topic make important contributions to our understanding of how livestock production can be improved with the application of various diagnostic and treatment options in clinical and comparative veterinary medicine. A plethora of future studies are proposed, suggesting that precision medicine, treatments with novel compounds, genetic improvement, nutritional manipulation, and adoption of advanced management practices could all be used to enhance the health status and productive performance of various livestock species in future. However, further research on these topics is required to develop an improved understanding of the use of alternative strategies for the treatment of various pathological disorders. The authors would like to thank all the contributors who participated in this Research Topic for their unwavering support.

## Author contributions

Both authors listed have made a substantial, direct, and intellectual contribution to the work and approved it for publication.
